# No implementation without cultural adaptation: a process for culturally adapting low-intensity psychological interventions in humanitarian settings

**DOI:** 10.1186/s13031-020-00290-0

**Published:** 2020-07-14

**Authors:** Camila Perera, Alicia Salamanca-Sanabria, Joyce Caballero-Bernal, Lya Feldman, Maj Hansen, Martha Bird, Pernille Hansen, Cecilie Dinesen, Nana Wiedemann, Frédérique Vallières

**Affiliations:** 1 Trinity Centre for Global Health, School of Psychology, Trinity College Dublin, Dublin, Ireland; 2 The IFRC Reference Centre for Psychosocial Support, Copenhagen, Denmark; 3 Health Department, Colombian Red Cross, Bogota, Colombia; 4 Department of Behavioural Science and Technology, Simon Bolivar University, Caracas, Venezuela; 5 ThRIVE, Department of Psychology, University of Southern Denmark, Odense, Denmark

**Keywords:** Cultural adaptation, Methodology, Mental health, Humanitarian settings, Refugees

## Abstract

**Background:**

Despite the widely recognised importance of cultural adaptation to increase the effectiveness of psychological interventions, there is little guidance on its *process*. Developed based on existing theory, we applied a four-step process to culturally adapt a low-intensity psychological intervention for use in humanitarian settings.

**Methods:**

The four-step process was applied to adapt a WHO low-intensity psychological intervention (i.e. Problem Management Plus, or PM+) for use with displaced Venezuelans and Colombians in Colombia. First, a rapid desk review was used as an (1) *information gathering* tool to identify local population characteristics. Next, the results were taken forward for the (2) formulation of *adaptation hypotheses*, whereby PM+ protocols were screened to identify components for adaptation, drawing on the Ecological Validity Model. Third, the elements flagged for adaptation were taken forward for (3) *local consultation* to firstly, verify the components identified for adaptation, to identify other areas in need of adaptation, and thirdly, to adapt the intervention protocols. Finally, the adapted intervention protocols were reviewed through (4) *external evaluations* with local experts.

**Results:**

The *information gathering* phase yielded key information on the socioeconomic aspects of the groups targeted for intervention, the availability and need for mental health and psychosocial support, and existing barriers to accessing care. The *adaptation hypotheses* phase further identified the need for clearer explanations of key concepts, the need for sensitive topics to match local attitudes (e.g., domestic violence, thoughts of suicide), and the identification of culturally appropriate social supports. Building on these first two phases, *local consultation* subsequently resulted in revised PM+ protocols. The adapted protocols differed from the original format in their focus on the problems unique to these population groups, the way that psychological distress is expressed in this context, and the inclusion of locally available supports. The results of the *external evaluation* supported the adaptations made to the protocols.

**Conclusion:**

The proposed four-step process offers a useful guide for *how* to adapt low-intensity psychological intervention within humanitarian settings. Despite some limitations, we show that even when time and resources are scarce it is possible and necessary to culturally adapt psychological interventions. We invite further testing, replication, and improvements to this methodology.

## Background

Psychological interventions, such as the Common Elements Treatment Approach, Problem Management Plus (PM+), and Narrative Exposure Therapy, are commonly implemented in humanitarian contexts as a way to reduce the negative impact of exposure to adversity and trauma [1]. *Cultural adaptation* of psychological interventions is therefore “the systematic modification of an evidence-based treatment or intervention protocol to consider language, culture, and context in such a way that is compatible with the client’s cultural patterns, meanings, and values” [2]. The importance of cultural adaptation has been demonstrated by a number of meta-analyses, which found that culturally adapted psychological interventions are associated with greater effectiveness than non-culturally adapted psychological interventions. One meta-analysis of 76 studies (25,225 participants) found a moderately strong effect of culturally adapted (*d* = .45) over unadapted psychological interventions [3]. Another meta-analysis of 65 studies (8620 participants) showed that culturally adapted psychological interventions were moderately more effective (*d =* .46) and that the most effective treatments were those with the largest number of adaptations [4]. Another meta-analysis of 21 studies comparing unadapted and culturally adapted psychotherapies found that culturally adapted interventions were modestly, but significantly more effective (*d =* .32) [5]. More recently, a meta-analysis showed a statistically significant benefit in favor of culturally adapted treatments of depressive disorders (SMD = − 0.72) delivered by mental health specialists and/or lay providers, compared to non-culturally adapted interventions [6].

Some have argued that adaptations should be conducted only when there is evidence that the original intervention does not fit the populations’ needs [7]. Others have questioned whether the additional costs of conducting cultural adaptations are justified by the superior clinical outcomes [8]. Culturally adapting interventions however, is also an ethical responsibility, as it reduces the risk of experiencing treatments or interventions that intrude or transgress individual cultural values and norms [9]. The greater effectiveness of culturally adapted interventions may therefore be explained by the increased acceptability, appropriateness, the client’s better understanding of the intervention as well as sustained fidelity [10, 11]. Correspondingly, implementing psychological interventions that have not been culturally adapted can have negative consequences on treatment continuation, lead to unintended harmful practices and distrust in mental health care [12]. At its best, failure to culturally adapt a psychological intervention could lead to inefficient use of scarce resources; at worst it could cause harm.

Despite its noted importance, there is a dearth of literature documenting the process of cultural adaptation of psychological interventions in a systematic way, both within and outside humanitarian contexts. Within humanitarian settings, working with vulnerable populations, limited funding and human resources, poor security and logistics, limit the capacity of providers to culturally adapt interventions [13]. As a result, and contrary to the agreed upon minimum guidelines on mental health and psychosocial support (MHPSS)^1^ in humanitarian settings [14], programme implementers often overlook cultural adaptation as a critical step in the implementation of MHPSS programming.

One early adaptation model, known as the Ecological Validity Model (EVM), has been recurrently applied to the development and adaptation of psychological interventions. The EVM proposes eight dimensions to guide cultural adaptations across language, persons, metaphors, content, concepts, goals, methods and context. The explicit adaptation of interventions across these eight dimensions is thought to increase the ecological and external validity of an intervention [11]. A second frequently cited model, the Cultural Sensitivity (CS) approach, originates from the field of substance use prevention research and recommends that changes are made to two possible structures within an intervention: the surface and deep structures [15]. The surface dimension involves matching the intervention’s content and messages to observable social and cultural behaviour. The second dimension is concerned with understanding how the cultural, social and historical environments influence health behaviour [15]. More recently, Heim and Kohrt [16] proposed a conceptual framework outlining specific elements to be adapted (i.e. cultural concepts of distress, treatment components and treatment delivery). Despite their influence, however, these theoretical approaches remain largely prefatory, lacking specific steps, or methodology, that describe *how* to culturally adapt an evidence-based intervention. Moreover, there is no evidence to suggest that one model of adaptation is superior to another [17].

Another model, known as the Heuristic Framework for Cultural Adaptation, proposes a frame of reference for conducting adaptations, which includes: (1) information gathering (2) preliminary adaptation design (3) preliminary adaptation tests, and (4) adaptation refinement [18]. A key characteristic of this process is that it allows the integration of dimensions, such as the ones outlined in the EVM in an operational and systematic way. Namely, this framework combines prescriptive and non-prescriptive approaches to cultural adaptations [10]. More recently, the DIME methodology offers a comprehensive roadmap on cultural adaptation for humanitarian organisations [19]. Though initially developed to assist researchers and practitioners in the implementation of mental health and HIV-related programmes, the DIME adaptation guidance offers important insight into how to adapt mental health interventions within humanitarian responses [19]. Among its features, the DIME methodology favours incorporating qualitative participatory techniques and suggests involving experts as well as community members in the process of cultural adaptation. However, the DIME methodology is time and labour intensive, requires several staff, and can take up to 8–10 months to complete. More practical processes, that retain a systematic approach, are therefore necessary for use within the more time-constrained and fast-paced contexts of humanitarian emergencies.

Notwithstanding the potential of existing models to inform the design of cultural adaptation methodologies, there is a need to describe how cultural adaptations can be implemented for psychological interventions within humanitarian contexts in a systematic and timely way [20]. To address this gap, we drew on the Heuristic Framework for Cultural Adaptation, while also incorporating useful elements of the DIME methodology and the Ecological Validity Model, to develop a systematic, four-step process to culturally adapt a low-intensity psychological intervention for use in humanitarian settings. This process is in line with the four overarching phases of cultural adaptation identified across a wide range of public health studies [21]. We illustrate this process through its application to the cultural adaptation of the World Health Organization (WHO)’s Problem Management Plus (PM+) intervention to Venezuelan migrants and refugees and Colombian Internally Displaced Persons (IDPs) living in Saravena, Colombia.

## Methods

### Setting

Colombia consistently ranks among the countries with the highest number of IDPs globally [24]. Today, internal displacement continues despite the 2016 Peace Agreement [25]. In addition, Colombia is currently the primary destination for migrants and refugees from neighbouring Venezuela [25]. At the time of writing, more than 1 million Venezuelans are estimated to have crossed the border to move to Colombia, fleeing Venezuela’s complex socioeconomic and political crisis at an approximate rate of 35,000 persons per day [26].

In response to the MHPSS needs of both Venezuelan migrants and IDPs, the Colombian Red Cross is currently implementing PM+ [27]. This research project arose out of a series of requests from Red Cross and Red Crescent National Societies for guidance on how to implement the World Health Organization’s low-intensity psychological interventions (including Problem Management Plus) for displaced populations. Problem Management Plus was specifically selected as it was developed for adults suffering from psychological distress (e.g., depression, anxiety, stress or grief), as well as a wide range of self-identified practical problems (e.g., unemployment, interpersonal conflict), all of which are prevalent among displaced populations. PM+ adopts aspects of the principal therapeutic approach (i.e. Cognitive Behavioural Therapy, or CBT^2^) to allow lay providers such as Colombian Red Cross volunteers to administer the intervention. The intervention and its core strategies (i.e. stress management, problem solving, behavioural activation and strengthening social support) are described in greater detail elsewhere (see: Dawson, Bryant, Harper, Kuowei Tay, Rahman, Schafer and van Ommeren [28]) and its scalability is currently being assessed across eight countries [29]. Multiple language versions of PM+ are available in the WHO’s website [27]. The PM+ manual instructs users to adapt the content to the local context to include a series of considerations (e.g., local expressions and metaphors, socio-cultural differences on how help is offered, differences in terms of locally available resources). PM+ has been found to be effective in reducing symptoms of psychological distress and post-traumatic stress disorder (PTSD), compared to enhanced treatment as usual across three randomised control trials [22, 23, 30].

As with all cultural adaptations, the aim of the present study was to identify what changes are required to the current version of the intervention’s protocols to make it more meaningful and acceptable to beneficiaries.^3^ To this end, four steps were applied to determine which change(s), if any, were required to the existing PM+ protocols and delivery for implementation with Venezuelan migrants and refugees and Colombian IDPs living in Saravena, Colombia. These steps are summarised in Table 1. Ethical approval for this study was provided by Trinity College Dublin (Dublin, Ireland) and CES University (Medellin, Colombia). Written consent forms were obtained from interviewed participants.

**Table 1:** A four-step process to culturally adapt low-intensity psychological interventions in humanitarian contexts

Steps	Description	Tools
Step 1: information gathering	Conduct a rapid desk review to gather relevant pre-existing information (e.g., demographic, socio-economic, help-seeking patterns, coping mechanisms)	Desk review guidance on MHPSS [31] Additional file 1
Step 2: adaptation hypotheses	Revise PM+ protocols to identify components for adaptation based on the Ecological Validity Model.	Eight-dimension matrix of the Ecological Validity Model [11] Table 2
Step 3: local consultations	Develop a focus group discussion guide based on Step 2 and use it to interview local specialists, community members and implementers to elaborate and/or validate previous findings.	A step-by-step guide to thematic analysis [32] Focus Group Discussion guide Additional files 2 and 3
Step 4: external evaluation	Engage two external reviewers in the evaluation of the PM+ intervention protocol using the Cultural Relevance Questionnaire to determine the protocol’s level of functional, conceptual and linguistic equivalence.	Cultural Relevance Questionnaire (CRQ) Additional file 4

### Step 1: information gathering

First, a rapid desk review of both peer-reviewed and grey literature (e.g., news articles, non-governmental organisation and government reports) was conducted over the course of 4 days by the lead author [33]. Demographic, socioeconomic, cultural and general health and mental health aspects of the intervention populations were gathered, according to the outline provided as Additional file 1. This outline was developed by the WHO and the United Nations Refugee Agency [31] and is designed to assist in drafting literature reviews that are immediately useful sources of information for practitioners in humanitarian settings. Features of this approach are that the review can be completed in as little or as much time as the nature of the emergency response (i.e. acute response period or protracted emergency) allows [34]. The process of designing the desk review guidance, and examples of completed desk reviews, are described elsewhere [34]. Among other information, this initial gathering stage sought to understand common problems among intervention populations, coping strategies, explanatory models for mental health and psychosocial problems and availability of mental health services. This information was then brought forward for Step 2 of the cultural adaptation process.

### Step 2: adaptation hypotheses

Next, PM+ intervention and training protocols were read and screened by the first author during 5 h to identify components of the intervention that could be subjected to cultural adaptation, across each of the eight EVM dimensions [11]. Possible sources of cultural non-fit (e.g., appropriate behaviour during sessions, taboo topics, somatic expressions of distress) were also identified. Available models (e.g., EVM, DIME methodology, CS) recommend adaptations to be informed by the expertise of stakeholders (e.g., practitioners, community members, implementers). Accordingly, possible sources of cultural non-fit identified in Step 2 were developed into specific questions to explore through focus group discussions. As such, formative research methods were applied to further understand the populations needs, risks and resilience factors [10, 17].

### Step 3: local consultation

Focus Group Discussions (FGDs) developed based on the results of the previous steps were then conducted with MHPSS specialists, programme implementers, and community members, consistent with the DIME methodology [19]. Of note, two FGDs were initially planned with representatives from the two intervention populations [11, 35] but unfortunately, security issues meant that these local consultations were cancelled. Thus, ultimately one FGD was conducted with four employees and volunteers from the Colombian Red Cross (Saravena branch), who were over 18 years of age, had roles as MHPSS volunteers and supervisors and worked closely with the intervention populations for the delivery of MHPSS programming. The FGD guide is presented as Additional file 2.

Local consultation lasted approximately 3 h and the audio was transcribed verbatim. Results were analysed using thematic analysis in NVivo (Version 11), following the steps or phases outlined by Braun and Clarke [32]. These steps are presented in Additional file 3. Thematic analysis was chosen due to its recognition as a flexible and useful method, that provides simplicity while also enabling a rich and detailed analysis of data [32]. The data was subjected to deductive analysis, whereby pre-determined topics and codes from step 2 (Table 2) were identified within the data. A second round of open-coding was conducted to allow for new codes to emerge. The resulting themes generated were subsequently used to inform the adaptation of the PM+ intervention and training protocols, as well as providers’ training and supervision, prior to having the culturally adapted version of the PM+ manual verified by external evaluators in Step 4.

**Table 2:** Adaptation hypotheses across the eight dimensions of the Ecological Validity Model

Dimensions	Adaptation hypotheses
Language	- Language should be simplified to match literacy level of providers and study populations
Persons	- Material’s graphics should depict individuals from both populations - Case examples should be adapted to reflect common problems and stressors, reactions and coping strategies among the population, as well as institutions and individuals providing support - Consider culturally appropriate interaction between providers and beneficiaries (e.g., physical contact, ways of addressing each other).
Metaphors	- Identify sayings and metaphors to express distress - Identify somatic expressions of psychological distress
Content	- Identify common and culturally appropriate social support and leisure activities - Consider culturally appropriate ways of discussing sensitive topics (e.g., domestic violence, thoughts of suicide, grief) - Intervention activities are understood and are appropriate in the cultural context
Concepts	- Key concepts (e.g., confidentiality, consent, intervention) and titles are correctly interpreted by both populations - Technical terms (e.g., distress, withdrawal, social isolation) match literacy level
Goals	- Intervention goals match social and cultural values
Methods	- Intervention delivered in a format that is acceptable to populations (e.g., including family, appropriate location, time between sessions) - Instruments are validated with target population and revised by volunteers for comprehension
Context	- Address barriers to participation (e.g., child-care, financial situation) - Address barriers for providers (e.g., time of day of sessions, reaching participant’s house) - Consider any issues unique to the populations context (e.g., family separation, migratory status) - Conduct sessions in culturally appropriate locations - Identify culturally and context appropriate referral pathways

### Step 4: external evaluations

The culturally adapted version of the PM+ intervention protocol was assessed by a Colombian and a Venezuelan psychologist, both based in academic institutions, who independently evaluated the protocol using the Cultural Relevance Questionnaire (CRQ), in approximately 3 h [36]. Grounded in cultural sensitivity and ecological validity theories and principles, the CRQ was specifically developed to evaluate the degree of ecological validity following cultural adaptation [2, 37]. A quick, easy to use, five-item questionnaire, the CRQ has demonstrated good reliability (Cronbach’s α = 0.74). The English-version of the CRQ is presented in Additional file 4. The CRQ also contains a section dedicated to the evaluation of each intervention module, or session of the intervention. This part of the CRQ was therefore divided into each of the five sessions of PM+ and these were independently evaluated according to content, case examples, and exercises.

## Results

What follows are the results of a systematic process for culturally adapting a low-intensity psychological intervention in humanitarian settings, described across four specific steps, and how applying these steps resulted in the cultural adaptation of PM+ for use among Venezuelans migrants and refugees and Colombian IDPs.

### Step 1: information gathering

Step 1 yielded practical information on the social and cultural context, including key barriers to accessing mental health care for Venezuelans and Colombian IDPs. Importantly, the information gathering stage elucidated how socioeconomic aspects, access to mental health and psychosocial support differed across these two population groups.

#### Socioeconomic aspects

Demographic data showed that approximately 33% of Colombian IDPs were children and young adults, with the majority not able to return to school following displacement [38]. Nearly a quarter (23%) of displaced Colombians had not gone to school [39]. Many did not have the education and training necessary for accessing the employment sector and unemployment was much higher among IDPs (76%), in comparison to the national rate of 12% [39]. Unemployment and employment in the informal sector were also higher among IDPs, which translates into economic instability and insecurity [40]. In contrast, 65% of Venezuelan migrants had reached secondary education and 29% had a technical or university degree [41]. A survey recently administered to newly regularised Venezuelans suggested that 46.3% of interviewees worked informally [42]. In addition to violence, sex work, theft, extortion, xenophobia, sleeping on the street, and risk of recruitment by armed groups or the drug trade, working dangerous jobs was a commonly reported threat among Venezuelan migrants and refugees [43].

#### Mental health and psychosocial support context

While a number of studies reported on the prevalence of mental illness among Colombian IDPs [44–48], information on the mental health of Venezuelan migrants remained largely anecdotal. A 2018 census of Venezuelans without legal residence in Colombia found that 3% of participants self-identified as having a mental health problem [49]. Overall, there was little literature offering explanatory models of mental health and psychosocial problems and help-seeking patterns for both study populations. A study identified domestic violence, unplanned pregnancies and mental health problems as frequently reported problems among displaced Colombians [40]. Drug addiction, domestic violence and mental health problems, including suicide attempts, were perceived as prevalent issues among young displaced women and men [40]. A study of 677 adults exposed to the armed conflict (including IDPs) on coping strategies found religion to be one of the most common used coping strategies among this population, together with “waiting for things to be fixed on their own” [50]. This same study found that adults exposed to the armed conflict were more likely to report religion as a coping strategy compared to other Colombians [50]. Conversely, qualitative research among women affected by the armed conflict (including IDPs) identified religion as an avoidance strategy [51].

#### Barriers to accessing mental health care

Both Venezuelan migrants and refugees and Colombian IDPs reportedly faced many barriers to accessing mental health care. For IDPs, these barriers include inability to pay for consultations, difficulties caused by bureaucratic requirements, discriminatory treatment and lack of transportation to and from health clinics [52]. Undocumented and unemployed Venezuelan migrants do not have access to the contributory system and therefore cannot access health insurance under the subsidised system [53]. Even though Colombian authorities have experience with internal displacement, this large transnational movement of people is reported to have led to major health and social challenges (e.g., difficulties in securing health care funding for migrants, regularisation of migrants) [53]. Their access is thus limited to emergency services and public health interventions offered by non-governmental organizations and government auxiliaries [53]. Among the main reasons given by Venezuelans for requesting temporary transit permits to Colombia were buying medicines and receiving medical care [54].

### Step 2: adaptation hypotheses

Table 2 demonstrates how the eight dimensions of the Ecological Validity Model [11] were elaborated across the components of PM+ for cultural adaptation, as informed by Step 1. The hypotheses described in Table 2 were subsequently formulated into open-ended questions for the FGD guide, as presented in Additional file 2. For example, as discussed in step 1, low education attainment is a common difficulty among Colombian IDPs leading to the identification of technical language as part of Step 2. Consequently, FGD participants were asked to read the original PM+ protocol before the FGD, asking them to pay particular attention to the language used in the manual and questions on terminology were probed during local consultation (Step 3). Similarly, Step 1 identified the lack of access to health and social services among Venezuelan migrants and refugees. In turn, the FGDs were developed to include a discussion on the services available to this population through government and non-governmental agencies.

### Step 3: local consultation

Results from the FGDs identified eight prominent themes for cultural adaptation of PM+ in this context.

#### Theme 1: promote social activities that support the community throughout the intervention

FGD questions centred on the types of support, resources and activities and yielded information on the importance of adapting the social activities presented in the PM+ intervention protocol to promote social cohesion and healthy habits within the community. For example, participants identified community members’ participation in community service days (e.g., cleaning the streets, opening ditches, changing or adding new pipes) as an important value and local resource. They acknowledged that while displaced Colombians are used to taking part in community work activities, Venezuelans have difficulties getting used to this practice. Participants considered contributing to community work an important factor in Venezuelans’ integration and acceptance by the community. Other identified social activities included teaching a skill or craft, such as playing the guitar, a new game or sewing, to children in the neighbourhood or in settlements. Participants highlighted the importance of including healthy living activities in the intervention manual (e.g., going to the river to swim during the weekends, going to the open-air gyms to exercise or joining an open-air gym class, dancing). These suggestions were subsequently integrated into case examples of the behavioural activation strategy (‘Get going, keep doing’) of PM+ as well as to the list of suggestions used for this strategy.

#### Theme 2: focus on common problems among Venezuelan migrants and refugees

When asked about common problems faced by both populations, participants discussed practical problems among Venezuelan migrants and refugees. These included economic needs, debts, lack of water in the settlements, family separation, overcrowding, threats of being expelled from the settlement if they do not contribute to the community work and recruitment by armed groups. Participants also reported how migrant children are bullied at school and that they have difficulties adapting to their new lives, as shown in the quote below by a CRC Volunteer:

*“I know fitting in has been hard for children … if something gets lost [at school] children would say ‘it was the Venezuelan child’, if there is a Venezuelan child, he would be the one to blame”*

These problems were used to reformulate case examples to be used during the training of CRC Volunteers. Table 3 shows examples of an original and an adapted case example.

**Table 3:** Original and adapted versions of a PM+ case example

Original version	37-year-old woman who witnessed her son killed in a motorbike accident 4 months ago. She is very anxious about the safety of her other children- she does not let them play outside anymore for fear they might be killed. She continues to grieve the loss of his son and finds it difficult to be around her other children because this makes her miss her son and she becomes very upset. When her children misbehave, she gets very angry with them and will tell them that the “good son” died. She feels irritable all the time and is now crying uncontrollably. She is embarrassed about this as it can happen when she is around her friends or at work.
Adapted version	Angela is a 32-year-old woman who arrived from Venezuela 3 months ago to look for work. She left her two children in Venezuela with her mother because she was worried about bringing them without having a place to stay. She must send them food and other products and she also has to pay her rent, but she hasn’t found a job. She worries about her children and her mother having difficulties and sometimes she can’t sleep through the night. There have been a few days when she feels that her situation is so desperate that she does not want to leave the house.

#### Theme 3: include common explanatory models of distress

While participants presented some explanatory models of mental health (e.g., family conflicts, professional challenges, challenges in their daily life), economic problems emerged as the most recurrent explanatory model of distress among IDPs and Venezuelan migrants and refugees, together with the situation of displacement. Participants also highlighted how ‘*being bored’* is commonly used among Venezuelans and Colombians alike to express withdrawal, and as a common expression of depression in this context. This expression along with the explanatory models of distress were therefore integrated into the psychoeducation (‘Understanding Adversity’) and problem-solving strategies (‘Managing Problems’) case examples.

#### Theme 4: map available social support networks

Participants listed the available support networks and services to both intervention populations (e.g., the CRC Listening Centre, other non-governmental organisations providing material support such as food vouchers or prosthetics and psychosocial support to migrants, refugees and IDPs, the local government entity in charge of protecting human rights). With this information, a mapping exercise was developed for training volunteers involved in the delivery of PM+. Volunteers were also trained on how to regularly map the services available, as these services are known to change constantly in humanitarian contexts. This would enable volunteers to better refer beneficiaries to appropriate organizations and services.

It was also found that Venezuelans tend to seek support and protection from other Venezuelans, while Colombians resort to neighbours for sharing their problems or distress. Some social networks were discussed as having negative influences (e.g., drinking every day with friends, joining armed groups). These examples were included in the training of CRC Volunteers for how to respond when beneficiaries propose negative coping strategy for managing their problems.

#### Theme 5: change images to show a more realistic picture of the person and include different groups

Participants discussed some of the visual changes required to the images in the PM+ intervention protocol. For example, they argued that more images of men were needed. In addition, participants requested more realistic images. Specifically, they requested that images portray clear and genuine expressions, as expressed by the supervisor:

*“It is important to highlight that the supporting images should not be disfigured or distorted, they should reflect the local context, and consider gender so beneficiaries feel represented in the pictures”*

**Fig. 1:**
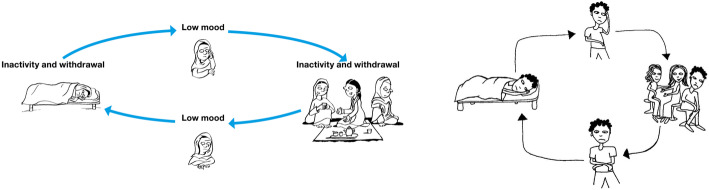
Original version of inactivity cycle of the PM+ behavioural activation strategy on the left and adapted version on the right

Participants also stressed the importance of representing different groups within the images (i.e. young and older adults, people with disabilities, indigenous people and Afro-Colombians). Images were therefore adapted to reflect the suggestions in themes 6 and 7, an example of which is provided in Fig. 1.

#### Theme 6: consider that community work acts as a source of conflict and distrust

Although no questions directly enquired about the relationship and interactions between Venezuelans and Colombians, participants recurrently talked about difficulties of the integration of Venezuelans and relations between both groups. Interviewees, who were all Colombian nationals, described how while Venezuelans were initially met with solidarity, distrust towards Venezuelans had developed, as illustrated in the quote below by one of the CRC Volunteers:

*“There has been a change. At the beginning, Colombians were more sympathetic, they were kinder to arrival of Venezuelans. However, there were reports of theft and murders of landlords renting to Venezuelans and there’s been many examples of these types of situations which have caused distrust and rejection”*

Examples of discrimination were also given, and it was suggested that Venezuelans have to adapt quickly to their new context, as one CRC Volunteer said:

*“They have had to adapt … and those that cannot, must leave the settlement due to the rules of coexistence of the settlement”*

Although the Colombian Red Cross branch representative had not received any reports of xenophobia, the interviewees noticed that Venezuelans were now less welcome in the community and suggested this was due to Venezuelan’s lack of familiarity with shared community systems. For example, in most communities, the water bill is shared among the neighbours, which is a system which may be unfamiliar to incoming migrants. Another is that communities hold community workdays every 8–15 days.

Despite difficulties, one of the participants talked about how Venezuelans are adapting to their communities. Participants recurrently presented how solidarity is valued by Colombians while Venezuelans were often perceived as rude and lazy, as said by a CRC Volunteer:

*“It also shows that we are from completely different cultures. We come from a culture where, be it good or bad, we have values that have been entrenched through by our families and they are sometimes very rude”*

These findings led to adding two exercises on subjectivity and objectivity to the training manual, to ensure that PM+ providers (i.e. CRC Volunteers) put personal values aside when providing PM+ to Venezuelan migrants and refugees. The exercises consisted of presenting various case examples where the narrator was being subjective or objective. Throughout these exercises the volunteers were asked to differentiate between the cases and discuss how the different ways of presenting the case would affect the interaction between intervention participants and volunteers and the type of care they receive.

#### Theme 7: change titles and terminology to match literacy level

Interviewees also proposed a series of changes to the titles of the sessions and strategies in the protocol, with the purpose of facilitating participants’, as well as providers’ understanding. For example, the word ‘adversity’ in title of the psychoeducation component ‘Understanding adversity’ and throughout the PM+ protocol was changed for ‘difficulties’, to facilitate understanding. Likewise, the title of the intervention (i.e. Problem Management Plus) was changed to ‘Managing your problems’ (‘Manejando tus problemas’). This was done to emphasize on the ownership beneficiaries have of their problems, to make sure volunteers remember that the problems discussed during the session are the beneficiary’s own difficulties, and in this way prevent volunteer burnout. Finally, the language used in the intervention protocol was considered too difficult for some volunteers, many of whom come from diverse professional and educational backgrounds (e.g., economist, farmer, nurse, engineer). Accordingly, the entire protocol was revised by the first author to ensure it matched the literacy level of all volunteers (e.g., replacing the terms meetings with sessions, questionnaires with forms, strategies with tools).

#### Theme 8: integrate good practices for volunteers’ relations with participants

A series of recommendations were made to improve the quality of the delivery of PM+. These recommendations were therefore added as an exercise to the PM+ training manual. Recommendations for improving the quality of service delivery included instructing lay providers on how to maintain a professional relationship (e.g., calling beneficiaries by their names, avoiding nicknames, ways of facing beneficiaries during sessions, not sharing their personal phone number with beneficiaries). In addition, for security reasons, it emerged that PM+ providers should go to the settlements in teams and to the PM+ sessions with a second volunteer. This was seen as a method to contribute to peer support and supervision within the implementation. This model would also allow for child-care in the instance when a parent was attending a PM+ session, such that the second volunteer could care for the children.

### Step 4: external evaluations

Across the categories, the cultural adaptation was scored as average to good (3.5 out of 5). Similarly, the adaptation across sessions was considered average to good (3.5 out of 5), with the first session of PM+ scoring the lowest and the third and fourth session scoring the highest. While mean scores are indicative of satisfactory cultural adaptation, the CRQ, more importantly, led to a number of additional recommendations for the cultural adaptation of PM+ for use in the Colombian context.

A suggestion was made to include real-life case studies of persons from the study populations containing commonly used cultural expressions when presenting the problem management strategy. Adding real-life case examples was seen as a way to contribute to the cultural sensitivity of the protocol and to the work of providers. It was also suggested to add more examples to the protocol’s session 2 to improve providers’ understanding of the strategies presented. It was also found that some literal translations of the content from English, the original language of the protocol, were incorrect, and recommendations were made for revisions of specific parts of the protocol. One of the reviewers highlighted the possibility that beneficiaries with lower levels of education might have some difficulties understanding certain concepts. Conversely, another reviewer argued that most concepts would be understood by Venezuelans and Colombians alike, but some national and regional cultural idioms might emerge during the sessions. Accordingly, it was suggested to recruit Venezuelan providers who can discern these cultural concepts.

One reviewer emphasised the importance of referring to women and men throughout all examples. This would be particularly important in the section describing sexual violence which presents the example of a woman, but which should also consider the perspective and circumstances of men. Another suggestion was to include more examples of the problem of discrimination towards migrants. Images were considered too general by one of the reviewers, who thought they did not reflect the specific characteristics of displaced Colombians. A final suggestion was to include additional culturally relevant activities in the description of the behavioural activation strategy such as cooking and preparing typical food (e.g., *hayacas* and *tamales)* as well as listening or dancing to popular songs (e.g. *vallenato*) to break the inactivity cycle.

## Discussion

Humanitarian MHPSS programming has previously been criticised for its lack of cultural sensitivity [55]. This has led to calls for clearer methods on how to contextualise MHPSS interventions [16, 56–58]. This study contributes towards bridging this gap by proposing and demonstrating a four-step process for culturally adapting low-intensity psychological interventions for use in humanitarian contexts. Cultural adaptation has primarily been an area of focus of academics and researchers. The process outlined in this study was therefore designed for practitioners or implementers without research experience. Although a qualitative analysis software was used to analyse the outcomes of the local consultations, the steps for thematic analysis can be followed manually using notes instead of transcripts and highlighters or coloured pens or Natural Language Processing [59]. While it is a systematic process, it is also meant to be simple, making it potentially feasible for a wide range of humanitarian responses, where interventions need to be implemented promptly, and time and human resources are scarce. This process can also be adjusted to incorporate new cultural adaptation tools as they emerge (e.g. FRAME) [60]. Another positive characteristic of this process is that, although it is presented as a stepped model, it can also be understood as an iterative process, as social and cultural constructs and understanding constantly evolve. Thus, when the emergency recedes it can be reapplied to consider new literature, new group dynamics as well as the experience gained by implementers through their work with these populations.

Several limitations of this methodology and its application in the present study should be acknowledged. We did not test whether this adapted version was more effective than a non-adapted version of PM+ in this context. Secondly, despite our intentions to include community consultations in step 3 of this methodology (i.e. local consultation), this was not possible due to the unstable security situation in the area. Implementation research in humanitarian settings must, first and foremost, respect the ethical principle of safety towards both participants and the research team, which could not be ensured at the time of the study [61]. However, excluding community members from this process implies that the adaptations made to the PM+ manuals reflect the opinions of professionals and not necessarily the direct perceptions, attitudes and cultural understandings of beneficiaries. We recommend practitioners experiencing similar challenges to consider the possibility of contacting community members for phone interviews. Lastly, the objective of cultural adaptations is to balance fit and fidelity, such that psychological interventions are supported by evidence, while also responding to the person’s culture and context. To achieve this, it is recommended to follow adaptation protocols or frameworks that support adaptations to language and context without altering the core intervention content [6, 57]. Furthermore, it is important to document the adaptation process to monitor changes that may affect fidelity [60]. Following these recommendations, however, does not guarantee that fidelity is maintained throughout this process. To further ensure that fidelity has not been compromised, future adaptations could request a review from a person with knowledge of the intervention, prior to implementation.

As case studies were adapted based on the outcomes of the local consultation (i.e. step 3), it is important to note that the person conducting this adaptation could have introduced their subjective bias. To further prevent this in the future, and if time and resources allow, cultural adaptations can also make use of other methods for adapting case studies. Some examples of these methods include: (1) cognitive interviewing for adapting case studies as described in the DIME methodology and as explained by Brown, et al. [62]; (2) presenting a focus group discussion with a blank template of how a case example should be structured so they can collaboratively develop an example based on experience; (3) developing case examples through the use of Forum Theatre or Theatre of The Oppressed [19, 63] or (4) employing elicitation techniques for cultural domain analysis [64]. A limitation of the fourth step of this process adaptation was the absence of a baseline measure of the Cultural Relevance Questionnaire. Future adaptations could consider including a baseline assessment of the protocol’s cultural sensitivity to further ascertain the impact of the cultural adaptation process.

## Conclusion

As psychological interventions, such as PM+, start to be implemented globally it is important to identify and test procedural frameworks for cultural adaptation. Combining aspects of various cultural adaptation frameworks and methodologies, we propose a four-step process for culturally adapting low-intensity psychological interventions for use in humanitarian contexts. Using a combination of a rapid literature review, systematic manual screening, stakeholder participation, and expert validation methods, we propose an ethical and appropriate method that can be carried out with minimal time and resources. Further reflections and accounts of the utility and effectiveness of this or similar processes are strongly encouraged.

## Supplementary information

**Additional file 1.** Template for Desk Review of Pre-Existing Information Relevant to Mental Health and Psychosocial Support in the Region/Country.

**Additional file 2.** Focus Group Discussion Guide for Local Consultation.

**Additional file 3.** Phases of Thematic Analysis (Reprinted with permission from authors) Source: Braun V, Clarke V. Using thematic analysis in psychology. Qual Res Psychol. 2006;3(2):77–101.

**Additional file 4.** Cultural Relevance Questionnaire (CRQ) English version.

## Data Availability

Please contact author for data requests.

## Abbreviations

CRC: Colombian Red Cross
CS: Cultural Sensitivity Approach
EVM: Ecological Validity Model
FGD: Focus Group Discussion
IDP: Internally Displaced Person
MHPSS: Mental Health and Psychosocial Support
PTSD: Post-Traumatic Stress Disorder
PM+: Problem Management Plus
WHO: World Health Organization

## Footnotes

1 Mental health and psychosocial support (MHPSS) is a term commonly used by international non-governmental organisations to describe the provision of a range of services to alleviate distress and support the mental health and wellbeing of both the individual and the community.

2 Cognitive behavioural therapy is one of the most researched forms of psychotherapy. The basic principle behind CBT is that thoughts, feelings and behaviour are all closely connected and have a decisive influence on our wellbeing.

3 Beneficiary is a term commonly used by non-governmental organisation implementers to refer to the persons that receive their services.
