# Supplementary material for: No implementation without cultural adaptation: a process for culturally adapting low-intensity psychological interventions in humanitarian settings
Source: Confl Health. 2020 Jul 14;14:46. doi: 10.1186/s13031-020-00290-0 (PMC7362525; doi:10.1186/s13031-020-00290-0)
Supplement: Supplementary file 1 — Additional file 1. Template for Desk Review of Pre-Existing Information Relevant to Mental Health and Psychosocial Support in the Region/Country. [file 13031_2020_290_MOESM1_ESM.docx]

Additional file 1. Template for Desk Review of Pre-Existing Information Relevant to Mental Health and Psychosocial Support in the Region/Country

Source: World Health Organization, United Nations High Commissioner for Refugees. Assessing mental health and psychosocial needs and resources: toolkit for humanitarian settings. Geneva: World Health Organization; 2012.

| Sample Table of Contents of a Literature review |
| --- |
| 1. Introduction  1.1. Rationale for the desk review (description of current/recent  emergency)  1.2. Description of methodology used to collect existing information  (including any database search terms used)  2. General Context  2.1. Geographical aspects (e.g., climate, neighboring countries)  2.2. Demographic aspects (e.g., population size, age distribution,  languages, education/literacy, religious groups, ethnic groups, migration patterns, groups especially at risk to suffer in humanitarian crises)  2.3. Historical aspects (e.g., early history, colonization, recent political history)  2.4. Political aspects (e.g., organization of state/government, distribution of power, contesting sub-groups or parties)  2.5. Religious aspects (e.g., religious groups, important religious beliefs and practices, relationships between different groups)  2.6. Economic aspects (e.g., Human Development Index, main livelihoods and sources of income, unemployment rate, poverty, resources)  2.7. Gender and family aspects (e.g., organization of family life, traditional gender roles)  2.8. Cultural aspects (traditions, taboo, rituals and practices related to health and well-being)  2.9. General health aspects  2.9.1. Mortality, threats to mortality, and common diseases 2.9.2. Overview of structure of formal, general health system  3. Mental Health and Psychosocial Context  3.1. Mental health and psychosocial problems and resources  3.1.1. Epidemiological studies of mental disorders and risk/ protective factors conducted in the country, suicide rates  3.1.2. Local expressions (idioms) for distress and folk diagnoses,  local concepts of trauma and loss  3.1.3. Explanatory models for mental and psychosocial problems  3.1.4. Concepts of the self/person (e.g., relations between body,  soul, spirit)  3.1.5. Major sources of distress (e.g., poverty, child abuse, infertility)  3.1.6. Role of the formal and informal educational sector in  psychosocial support  3.1.7. Role of the formal social sector (e.g., social services) in  psychosocial support  3.1.8. Role of the informal social sector (e.g., community protection  systems, neighborhood systems, other community resources)  in psychosocial support  3.1.9. Role of the non-allopathic health system (including traditional  or indigenous health system) in mental health and psychosocial  support  3.1.10. Help-seeking patterns (where people go for help and for  what problems; who accompanies them; potential barriers  to access)  3.2. The Mental Health System  3.2.1. Mental health policy and legislative framework and leadership  3.2.2. Description of the formal mental health services (primary, secondary and tertiary care). Consider the relevant Mental  Health Atlas and WHO-AIMS reports among other sources to find out availability of mental health services, mental health human resources, how mental health services are used, how accessible mental health services are (for example distance, fee for service), and the quality of mental health services  3.2.3. Relative roles of government, private sector, NGOs, and traditional healers in providing mental health care  4. Humanitarian Context  4.1. History of humanitarian emergencies in the country  4.2. Experiences with past humanitarian aid in general  4.3. Experiences with past humanitarian aid involving mental health  and psychosocial support  5. Conclusion  5.1. Expected challenges and gaps in mental health and psychosocial  support  5.2. Expected opportunities in mental health and psychosocial support  6. References |
