# Supplementary material for: No implementation without cultural adaptation: a process for culturally adapting low-intensity psychological interventions in humanitarian settings
Source: Confl Health. 2020 Jul 14;14:46. doi: 10.1186/s13031-020-00290-0 (PMC7362525; doi:10.1186/s13031-020-00290-0)
Supplement: Supplementary file 2 — Additional file 2. Focus Group Discussion Guide for Local Consultation. [file 13031_2020_290_MOESM2_ESM.docx]

Additional file 2. Focus Group Discussion Guide for Local Consultation

| Questions and probes |
| --- |
| PM+ will be administered to migrants and displaced persons, what are the best ways to let these populations know about the intervention?  Where should we disseminate information about PM+?  In your opinion, should information on PM+ be presented in any community meetings? If yes, to whom would be these presentations directed?  What in your experience are ways in which these populations express distress? Would you note any difference among certain sub-groups?  What type of behaviour would you observe among members of these populations when they are feeling distressed? Would you note any difference among certain sub-groups?  What are some expressions used to express sadness or stress? Would you note any difference among certain sub-groups?  In general, to what would people in this two populations attribute these feelings of sadness or anxiety? Would you note any difference among certain sub-groups?  What do people from these populations do to take care of their wellbeing? Would you note any difference among certain sub-groups?  What do people in these populations do to take care of their relatives and friends? Would you note any difference among certain sub-groups? |
| What do people from these two populations do to enjoy themselves?  What do some activities people from these two populations do to connect with others?  What do some activities people from these two populations do to take care of how they look and for self-care?  What do some activities people from these two populations do to be active?  What would be some activities people from these two populations do daily (e.g., cook, child-care)?  What are some activities people from these two populations do to support or help others (e.g., family, friends, neighbours)?  What types of support do people from these two populations have access to?  Which specific services are available to these two populations?  Which social services do Venezuelan migrants and refugees have access to?  Which organizations, community groups or community members are known for providing psychosocial support to members of these two populations?  What is the role of religious organizations in providing support (e.g., psychosocial, financial) to members of these two populations?  Can you list problems displaced persons have mentioned to you?  Can you list problems Venezuelan migrants and refugees have mentioned to you?  The PM+ manual provides some advice on how to manage sensitive topics (e.g., grief and loss, sexual abuse, ongoing threat). When revising these parts did you note down possible adaptations?  What social and cultural norms do volunteers need to consider when working with members of these two populations?  How should volunteers address the persons receiving the interventions?  Considering the cultural and social background of this communities, when and where should sessions take place?  How is physical contact viewed in these communities? |
| Considering the cultural and social background of these two groups, how should the concept of confidentiality be introduced?  Considering the cultural and social background of these two groups, how should the concept of consent be introduced?  Now let’s revise the titles of the manual and of the PM+ strategies, do you suggest any adaptations so they are more acceptable to members of these populations?  When revising the intervention, did you identify any terms or concepts that could be misunderstood or difficult to understand in this context?  Now I want you to think of a moment, when you provided words (incl. phrases and sayings) of support or encouragement to someone, what did you say?  What words (incl. phrases and sayings) of support or encouragement would someone from Venezuela use?  Now let’s revise the images in the manual.  Questions for each image:  Is this image acceptable (not offensive) among these two communities?  Does this image portray members of these populations?  What changes are necessary to make this image more relevant to the person receiving the intervention? |
