# Supplementary material for: No implementation without cultural adaptation: a process for culturally adapting low-intensity psychological interventions in humanitarian settings
Source: Confl Health. 2020 Jul 14;14:46. doi: 10.1186/s13031-020-00290-0 (PMC7362525; doi:10.1186/s13031-020-00290-0)
Supplement: Supplementary file 3 — Additional file 3. Phases of Thematic Analysis (Reprinted with permission from authors) Source: Braun V, Clarke V. Using thematic analysis in psychology. Qual Res Psychol. 2006;3(2):77–101. [file 13031_2020_290_MOESM3_ESM.docx]

Additional file 3. Phases of Thematic Analysis (Reprinted with permission from authors)

Source: Braun V, Clarke V. Using thematic analysis in psychology. Qual Res Psychol. 2006;3(2):77–101.

| Phase | Description of the process |
| --- | --- |
| 1. Familiarising yourself with your data | Transcribing data (if necessary), reading and re-reading the data, noting down initial ideas. |
| 2. Generating initial codes | Coding interesting features of the data in a systematic fashion across the entire data set, collating data relevant to each code. |
| 3. Searching for themes | Collating codes into potential themes, gathering all data relevant to each potential theme. |
| 4. Reviewing themes | Checking in the themes work in relation to the coded extracts (Level 1) and the entire data set (Level 2), generating a thematic ‘map’ of the analysis. |
| 5. Defining and naming themes | Ongoing analysis to refine the specifics of each theme, and the overall story the analysis tells; generating clear definitions and names for each theme. |
| 6. Producing the report | The final opportunity for analysis. Selection of vivid, compelling extract examples, final analysis of selected extracts, relating back of the analysis to the research question and literature, producing a scholarly report of the analysis. |
