# Supplementary material for: No implementation without cultural adaptation: a process for culturally adapting low-intensity psychological interventions in humanitarian settings
Source: Confl Health. 2020 Jul 14;14:46. doi: 10.1186/s13031-020-00290-0 (PMC7362525; doi:10.1186/s13031-020-00290-0)
Supplement: Supplementary file 4 — Additional file 4. Cultural Relevance Questionnaire (CRQ) English version. [file 13031_2020_290_MOESM4_ESM.docx]

**Cultural Relevance Questionnaire (CRQ)**

**English version**

Salamanca-Sanabria, A., Richards, D., Timulak, L (2016)

The Cultural Relevance Questionnaire is an instrument that evaluates a culturally adapted psychotherapy protocol. This questionnaire is based on cultural sensitivity and ecological validity theory by Bernal (2009), and Helms’ (2015) proposals for culturally evidence-based practices.

This questionnaire assesses a culturally adapted psychotherapy (CAP). CAP is defined as a systematic change of intervention protocols through which consideration of culture and context modifies treatment in accordance with clients’ values and contexts, relevant to the culture of the target population ([Bernal, Jiménez-Chafey, & Domenech Rodríguez, 2009](#_ENREF_12)).

CRQ is divided into three categories:

**Functional equivalence:** It is defined as the extent to which the same ostensible behaviours (e.g., crying) are interpreted similarly in different cultural or racial groups, occur with equal frequency within these groups, and elicit similar reactions from other members of the groups. [Components of the internet-delivered programme could be interpreted similarly by the target cultural group (e.g., personal stories, examples)].

**Conceptual equivalence**: refers to the extent to which different concepts are analogous for the cultural group that is targeted for the treatment. [Cultural expressions of depression, ideas or analogies about mental illness are included in the internet-delivered programme (e.g., symbols, metaphors and concepts)].

**Linguistic equivalence**: indicates the language or dialect used during the process and in evaluations of the process and outcome have been adjusted so that it has meaning to the person(s) being assessed. [Level of oral and written language adjustments are made for the internet-delivered programme (e.g. regionalism, slang)].

Likewise, cultural relevance is based on eight (8) areas descripted by Bernal (2009) for making culturally adapted psychotherapy, which are:

**Language**: Includes oral and written language, which must be culturally appropriate and syntonic, taking into consideration differences in inner city, regional or subcultural groups.

**Person(s):** Refers to the client–therapist relationship during the intervention.

**Metaphors:** Meaning to the symbols and concepts that are shared by a particular cultural group.

**Contents**: Refers to cultural knowledge about values, customs, and traditions shared by ethnic and minority groups.

**Concepts:** Indicate the constructs of the theoretical model to be used in treatment.

**Goals:** Implies the establishment of an agreement between the therapist and client as to the goals of treatment.

**Methods:** Refers to the procedures to follow for the achievement of the treatment goals.

**Contexts:** Indicates the consideration of the client’s broader social, economic, and political context.

**Cultural Relevance Questionnaire (CRQ)**

The general evaluation of cultural relevance treatment consists of three categories, which are explained below. Please assess these from 1 to 5 and explain your score.

**Categories:**

***Section 1. Functional equivalence***

The Functional Relevance of a questionnaire item refers to whether the item describes behaviour (example: depression) in a way that is interpreted similarly by your culture (example: is a crying person viewed and interpreted the same way in your culture?)

Please use this legend to answer the questions:

| 1  The components are not reflected within the programme. | 2  Most of the components are not reflected within the programme; however, some of them are. | 3  Some components are reflected within the programme and others are not. | 4  Most of the components are reflected within the programme; however, others are not. | 5  All of the components are reflected within the programme. |
| --- | --- | --- | --- | --- |

**For each question, please choose the response which best characterises the treatment assessed.**

- 1. The programme involves **behavioural** or **emotional expressions** familiar to the cultural group being targeted.

| 1 | 2 | 3 | 4 | 5 |
| --- | --- | --- | --- | --- |

| Please explain your choice |
| --- |

- 1. **The people** and **cultural context** are reflected in the treatment (e.g. social, political, economic, ethnic, historical).

| 1 | 2 | 3 | 4 | 5 |
| --- | --- | --- | --- | --- |

| Please explain your choice |
| --- |

1.3. The **treatment goals** are tailored to work with the user from this cultural context (e.g. examples, personal stories).

| 1 | 2 | 3 | 4 | 5 |
| --- | --- | --- | --- | --- |

| Please explain your choice |
| --- |

***Section 2. Conceptual equivalence***

The Conceptual Relevance of a questionnaire item refers to whether the item measures the same concept in your culture. It is the analogy grade is shared by a cultural group, such as: behaviours, symbols, metaphors and concepts. Assess this category on the programme in: psychoeducation sections, personal stories, examples, activities, imagens and quotes.

2.1 The treatment includes **symbols** and **concepts** shared by the cultural group, for instance cultural expressions of depression, ideas or analogies about mental illness are included in the program.

| 1 | 2 | 3 | 4 | 5 |
| --- | --- | --- | --- | --- |

| Please explain your choice |
| --- |

***Section 3. Linguistic equivalence:***

Involves oral and written language on the programme. Assess language on the programme, content, examples and activities

3.1 The treatment includes **written** and **oral communication** that can be considered dialects and jargon relevant in this cultural context (e.g. regionalism, slang).

| 1 | 2 | 3 | 4 | 5 |
| --- | --- | --- | --- | --- |

| Please explain your choice |
| --- |

**Culturally adapted treatment per module**

Please assess from 1 to 5 the functional relevance, conceptual relevance and linguistic relevance of each module on the programme:

***Functional equivalence*:** The Functional relevance of a questionnaire item refers to whether the item describes behaviour (example: depression) in a way that is interpreted similarly by your culture (example: is a crying person viewed and interpreted the same way in your culture?). Assess this category on the personal stories, examples.

***Conceptual equivalence****:* refers the analogy grade is shared by a cultural group, such as: behaviours, symbols, metaphors and concepts. Assess this category on the programme in: psychoeducation sections, activities, imagens and quotes.

**Linguistic *equivalence*:** Involves oral and written language on the programme. (Example: regionalism, slangs).

Please use this legend to answer the questions:

| 1  The components are not reflected within the module. | 2  Most of the components are not reflected within the module; however, some of them are. | 3  Some components are reflected within the module and others are not. | 4  Most of the components are reflected within the module; however, others are not. | 5  All of the components are reflected within the module. |
| --- | --- | --- | --- | --- |

**For each question, please choose the response which best characterises the treatment assessed.**

**Module 1:**

| **Components** | **Functional equivalence (1-5)** | **Conceptual equivalence (1-5)** | **Linguistic equivalence (1-5)** | **Observations** |
| --- | --- | --- | --- | --- |
| Content |  |  |  |  |
| Personal Stories |  |  |  |  |
| Examples |  |  |  |  |
| Activities |  |  |  |  |
| **General comments of the module** | | | | |

**Module:**

| **Components** | **Functional equivalence (1-5)** | **Conceptual equivalence (1-5)** | **Linguistic equivalence (1-5)** | **Observations** |
| --- | --- | --- | --- | --- |
| Content |  |  |  |  |
| Personal Stories |  |  |  |  |
| Examples |  |  |  |  |
| Activities |  |  |  |  |
| **General comments of the module** | | | | |

**Module:**

| **Components** | **Functional equivalence (1-5)** | **Conceptual equivalence (1-5)** | **Linguistic equivalence (1-5)** | **Observations** |
| --- | --- | --- | --- | --- |
| Content |  |  |  |  |
| Personal Stories |  |  |  |  |
| Examples |  |  |  |  |
| Activities |  |  |  |  |
| **General comments of the module** | | | | |

**Module:**

| **Components** | **Functional equivalence (1-5)** | **Conceptual equivalence (1-5)** | **Linguistic equivalence (1-5)** | **Observations** |
| --- | --- | --- | --- | --- |
| Content |  |  |  |  |
| Personal Stories |  |  |  |  |
| Examples |  |  |  |  |
| Activities |  |  |  |  |
| **General comments of the module** | | | | |

**Module:**

| **Components** | **Functional equivalence (1-5)** | **Conceptual equivalence (1-5)** | **Linguistic equivalence (1-5)** | **Observations** |
| --- | --- | --- | --- | --- |
| Content |  |  |  |  |
| Personal Stories |  |  |  |  |
| Examples |  |  |  |  |
| Activities |  |  |  |  |
| **General comments of the module** | | | | |

**Module:**

| **Components** | **Functional equivalence (1-5)** | **Conceptual equivalence (1-5)** | **Linguistic equivalence (1-5)** | **Observations** |
| --- | --- | --- | --- | --- |
| Content |  |  |  |  |
| Personal Stories |  |  |  |  |
| Examples |  |  |  |  |
| Activities |  |  |  |  |
| **General comments of the module** | | | | |

**Module:**

| **Components** | **Functional equivalence (1-5)** | **Conceptual equivalence (1-5)** | **Linguistic equivalence (1-5)** | **Observations** |
| --- | --- | --- | --- | --- |
| Content |  |  |  |  |
| Personal Stories |  |  |  |  |
| Examples |  |  |  |  |
| Activities |  |  |  |  |
| **General comments of the module** | | | | |

**Note:** The interaction on the platform and modules will be reviewed as part of the validity of this evaluation.
